# Patterns and predictors of antimicrobial resistance among *Staphylococcus* spp. from canine clinical cases presented at a veterinary academic hospital in South Africa

**DOI:** 10.1186/s12917-017-1034-3

**Published:** 2017-04-28

**Authors:** Daniel N. Qekwana, James W. Oguttu, Fortune Sithole, Agricola Odoi

**Affiliations:** 1 Section of Veterinary Public Health, Department of Paraclinical Sciences, Faculty of Veterinary Science, University of Pretoria, Pretoria, South Africa; 2 Department of Agriculture and Animal Health, College of Agriculture and Environmental Sciences, University of South Africa, Johannesburg, South Africa; 3 Ross University School of Veterinary Medicine, Basseterre, Saint Kitts and Nevis; 4 Biomedical and Diagnostic Sciences, College of Veterinary Medicine, University of Tennessee, Knoxville, USA

**Keywords:** *Staphylococcus aureus*, *Staphylococcus pseudintermedius*, multidrug resistance, MDR, Antimicrobial resistance, AMR, dogs, canine, predictors, risk factors

## Abstract

**Background:**

Antimicrobial resistance in staphylococci, often associated with treatment failure, is increasingly reported in veterinary medicine. The aim of this study was to investigate patterns and predictors of antimicrobial resistance among *Staphylococcus* spp. isolates from canine samples submitted to the bacteriology laboratory at the University of Pretoria academic veterinary hospital between 2007 and 2012. Retrospective data of 334 *Staphylococcus* isolates were used to calculate the proportion of samples resistant to 15 antimicrobial agents. The Cochran-Armitage trend test was used to investigate temporal trends and logistic regression models were used to investigate predictors of antimicrobial resistance in *Staphylococcus aureus* and *Staphylococcus pseudintermedius*.

**Results:**

Results show that 98.2% (55/56) of the *S. aureus* isolates were resistant to at least one drug while 42.9% were multidrug resistant. Seventy-seven percent (214/278) of the *S. pseudintermedius* isolates were resistant to at least one drug and 25.9% (72/278) were multidrug resistant. Resistance to lincospectin was more common among *S. aureus* (64.3%) than *S. pseudintermedius* (38.9%). Similarly, resistance to clindamycin was higher in *S. aureus* (51.8%) than *S. pseudintermedius* (31.7%) isolates. There was a significant (*p* = 0.005) increase in *S. aureus* resistance to enrofloxacin over the study period. Similarly, *S. pseudintermedius* exhibited significant increasing temporal trend in resistance to trimethoprim-sulphamethoxazole (*p* = 0.004), clindamycin (*p* = 0.022) and orbifloxacin (*p* = 0.042). However, there was a significant decreasing temporal trend in the proportion of isolates resistant to doxycycline (*p* = 0.041), tylosin (*p* = 0.008), kanamycin (*p* = 0.017) and amoxicillin/clavulanic acid (*p* = 0.032).

**Conclusions:**

High levels of multidrug resistance and the increasing levels of resistance to sulphonamides, lincosamides and fluoroquinolones among *Staphylococcus* spp. isolates in this study are concerning. Future studies will need to investigate local drivers of antimicrobial resistance to better guide control efforts to address the problem.

## Background

Resistance of *Staphylococcus* spp. organisms to antimicrobial agents used in dogs may complicate treatments and result in increased morbidity, mortality and financial burdens to the owners [1]. According to Werckenthin et al. [2], the prevalence of antimicrobial drug resistance in staphylococcal infections in dogs, cattle and pigs is on the rise. Prescott et al. [3], in their study conducted in Canada, observed that the majority of *S. intermedius* isolates were resistant to enrofloxacin and gentamycin. In another study by Hauschild and Wójcik [4] carried out in Poland, it was reported that 41% and 35% of *S. intermedius* isolates tested were resistant to erythromycin and clindamycin, respectively. In both studies, the authors attributed the high levels of resistance to changes in the pattern of antimicrobial use for treatment of staphylococcal infections. Moreover, Hauschild and Wójcik [4] as well as Pellerin et al. [5] suggest that rapid introduction and the over prescription of new antimicrobial drugs in companion animals may also be contributing to the increased antimicrobial resistance seen in staphylococcal organisms. Hoekstra and Paulton [6] also reported that the type of organism, site of isolation, sex and age of the dogs is associated with the risk of resistance.

In South Africa, 269,794 kg of parenteral antimicrobials were sold between 2002 and 2004, with penicillins being the most commonly (60%) sold antimicrobial followed by tetracyclines (32%) [7]. A study by Kudakwashe [8] on antimicrobial usage patterns among companion animal veterinarians in South Africa reported that the most commonly used antimicrobials by the respondents were cephalosporins (100%), followed by penicillins (98%), quinolones (95%), and lincosamides (52%). In addition, 28% of the respondents indicated that they did not undertake routine antibiograms in cases of therapeutic failures. This is surprising considering 81% of the respondents indicated that patients returned to the clinic due to treatment failures following use of the prescribed antimicrobials [8].

Although, several studies have reported increased antimicrobial resistance among *S. intermedius* isolates to ampicillin, penicillin and tetracycline [5, 9], not much information is available on antimicrobial resistance trends in *Staphylococcus* spp. in South Africa. Moreover, no work has been done to investigate the predictors of resistance among *Staphylococcus* spp. isolates from clinical cases. Therefore, the aim of this study was to investigate patterns and predictors of antimicrobial resistance among *Staphylococcus* spp. isolates from canine samples submitted to the bacteriology laboratory at the University of Pretoria academic veterinary hospital between 2007 and 2012.

## Methods

### Data collection and preparation

This was a retrospective study of *Staphylococcus* spp. isolates from canine clinical samples submitted to the University of Pretoria bacteriology laboratory as part of the routine diagnostic evaluation of cases presented to the university veterinary academic hospital between January 2007 and December 2012. All samples originated from cases treated at the university hospital. The bacteriology laboratory does not process samples from other sites/clinics/hospitals implying that samples included in the study are all from animals that were treated at the academic hospital. The data were assessed for duplicate entries, mixed infections (i.e. more than one isolate per sample), and if any animals were sampled multiple times during the study period. No duplicates were identified. Moreover, there were no samples with mixed infections (i.e. no samples with more than one isolate). Additionally, the dataset did not contain multiple tests from the same patient. The analyses were performed at isolate-level. However, since each sample had only one isolate, it implies that the results of isolate-level analysis are the same as sample level analysis (i.e. no problem of clustering arises).

A total of 334 confirmed *Staphylococcus* isolates consisting of *S. aureus* or *S. pseudintermedius* isolates were included in this study. Each case included in the analysis had data on the following variables: site of collection, breed, sex, age, date of submission and the antimicrobial agents tested. The American Kennel Club (AKC) breed classification was modified and used to classify breeds of dogs into the following categories: working, sporting, herding, hound, toy, terrier, nonsporting and mix-breed (http://www.akc.org/dog-breeds/). *Staphylococcus* species were identified based on the phenotypic characteristics including colony characteristics, catalase, D-mannitol, deoxyribonuclease (DNase) tests, and Gram-staining as described by Quinn et al. [10].

### Antimicrobial susceptibility testing

*Staphylococcus* isolates were subjected to antimicrobial susceptibility testing against a panel of 15 drugs using the disc diffusion method. The following antimicrobials were included in the panel: 30 μg amikacin (AK), 30 μg doxycycline (DOX30), 5 μg enrofloxacin (ENR), 10 μg gentamicin (CN), 10 μg ampicillin (AM) 10 μg penicillin G (P), 25 μg trimethoprim-sulphamethoxazole (co-trimoxazole) (SXT), 30 μg chloramphenicol (C), 30 μg cephalothin (KF), 30 μg kanamycin (K), 2 μg clindamycin (MY), 100 μg lincospectin (LS100), 5 μg orbifloxacin (OBX5), 20/10 μg amoxicillin/clavulanic acid (AMC20/10) and 15 μg tylosin (TY). Unfortunately, since the laboratory does not routinely assess for methicillin susceptibility, the panel did not include this drug and therefore this study was not able to investigate the resistance of *Staphylococcus* spp. to methicillin. The laboratory from where the data were obtained, follows the Clinical and Laboratory Standards Institute (CLSI) procedures [11–17] for isolation, testing and classification to determine the susceptibility profile (Susceptible, Intermediate or Resistant) of *Staphylococcus* isolates. The original raw data with exact diameter measurements of the inhibition zones were not available for this retrospective study. Thus, only interpretations of the susceptibility test results (i.e., Susceptible, Intermediate or Resistant) were available. Therefore, although a newer version of the CLSI document [18] is currently available it was not possible to interpret the susceptibility profile of isolates using this newer version of the document. For the purposes of the study, intermediate susceptibility was considered as susceptible and therefore re-coded as such for all subsequent analyses.

### Data analysis

All the statistical analyses were performed using SAS 9.4 (SAS Institute Inc., Cary, NC, USA) statistical package. The data were assessed for missing data and inconsistencies such as improbable values. Age was categorised into <2 years, 2–4 years, 4–6 years, 6–8 years and >8 years. The frequencies and proportions of all categorical variables together with their 95% confidence intervals were calculated. Associations between categorical variables were assessed using the Chi-square or Fishers Exact tests where appropriate. The Cochran–Armitage trend tests were used to assess temporal trends in the proportion of samples resistant to each antimicrobial agent between 2007 and 2012. Statistical significance was assessed at 5% level of significance for all the above tests.

Investigation of the predictors of antimicrobial resistance (resistance to at least one antimicrobial) was performed in two steps. In the first step simple binary logistic regression models were fitted with antimicrobial resistance status (yes/no) as the outcome and each of the suspected predictors available in the dataset (age, sex, breed) as the explanatory variables. For each of the simple binary logistic regression models, the predictor variables with *p*-values less than 0.20 were considered for inclusion in the multivariable logistic regression model fit in the second step of the modeling effort. In this second step, a multivariable logistic regression model using manual backwards selection was fitted containing all variables that had potential univariate associations (*p* < 0.2) with the outcome. Confounding was assessed by comparing the change in parameter estimate of the variables in the model with and without the suspected confounding variable. If there was a 20% change in the estimate, the variable of interest was considered to be a significant confounder and was retained in the final model. The odds ratios and their corresponding 95% confidence intervals were computed for all variables included in the final model. The predictor variables with *p*-values <0.05 were considered to be statistically significant based on the Wald Chi-Squared Test. Steps 1 and 2 above were repeated with binary (yes/no) multi-drug resistance (resistance to three or more antimicrobial classes) as the outcome variable. Goodness of fit of the final models was assessed using the Hosmer-Lemeshow goodness of fit test.

## Results

### Antimicrobial resistance patterns

Of the 1497 samples tested, 26.5% (396/1497) were *Staphylococcus* positive and included *S. aureus* (*n* = 57), *S. epidermidis* (*n* = 11), *S. pseudintermedius* (*n =* 284), *Staphylococcus*. spp. (*n* = 43) and *S. felis* (*n* = 1). Seven isolates [*S. pseudintermedius* (*n* = 6) and *S. aureus* (*n* = 1)] were not included in subsequent analyses due to missing information. Therefore, this study focuses only on the 334 samples that were positive for S. *pseudintermedius* (*n* = 278) or *S. aureus* (*n* = 56) and that did not have missing information. Twenty-five different types of specimens tested positive for *Staphylococcus* spp. with the skin contributing the most samples (34.4%, 115/334), followed by ear canal (29.9%, 100/334) while the rest of the 23 different specimen types making up the remaining 35.6% (119/334).

**Fig. 1:**
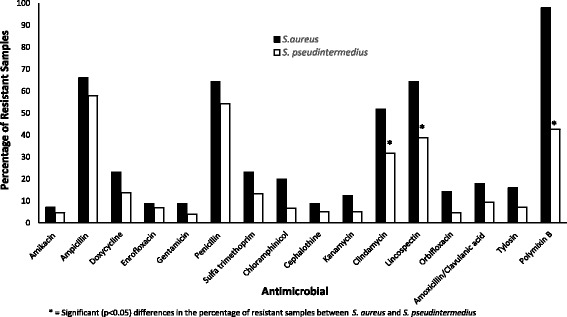
Proportions of *S. aureus* and *S. pseudintermedius* samples resistant to each of the 15 antimicrobials tested at the University of Pretoria bacteriology laboratory, 2007 and 2012

Over 50% of the *S. aureus* isolates were resistant to ampicillin (66.1%), penicillin (64.3%), lincospectin (64.3%), and clindamycin (51.8%). Similarly, over 50% of the S. *pseudintermedius* isolates were resistance to ampicillin (57.9%) and penicillin (54.3%) (Fig. 1). In addition, significant differences were observed in the proportion of *S. pseudintermedius* and *S. aureus* resistance to lincospectin (*p* = 0.0006) and clindamycin (*p* = 0.0055) (Fig. 1). In both instances, the proportion of *S. aureus* isolates were significantly higher than those of *S. pseudintermedius* isolates. These results show that the level of resistance among *Staphylococcus* isolates from dogs presented at the teaching hospital was very high, and also showed that *S. aureus* tended to exhibit higher resistance levels to certain antimicrobials compared to *S. pseudintermedius.*

### Temporal patterns in resistance patterns of *S. aureus* and S*. pseudintermedius*

**Fig. 2:**
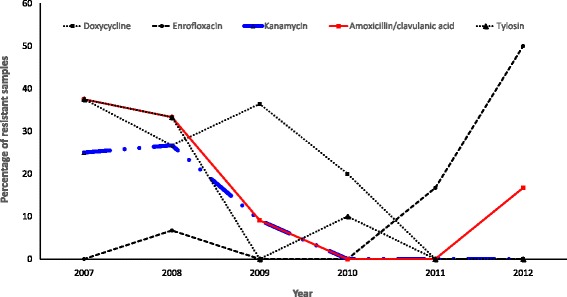
Antimicrobial agents showing significant temporal trends in resistance based on the Cochran-Armitage trend tests among the *S. aureus* isolates from canine samples tested at the University of Pretoria bacteriology laboratory, 2007–2012

The proportions of *S. aureus* isolates resistant to the different antimicrobials is shown in Table 1. Cochran-Armitage trend test showed that the proportion of *S. aureus* isolates that were resistant to doxycycline (*p* = 0.0412) or tylosin (*p* = 0.0083) significantly decreased from 37.5% in 2007 to 0.0% in 2012, while the proportion resistant to kanamycin significantly (*p* = 0.0167) decreased from 26.7% in 2008 to 0.0% in 2012. Similarly, a significant (*p* = 0.0317) decrease (37.5 to 0%) in the proportion of *S. aureus* isolates resistant to amoxicillin/clavulanic acid was observed between 2007 and 2011. However, increasing levels of resistance among *S. aureus* isolates to amoxicillin/clavulanic acid emerged by the end of 2012. A significant (*p* = 0.0052) increase in the level of resistance to enrofloxacin among *S*. *aureus* isolates was also observed between 2007 and 2012 (Fig. 2).

**Table 1:** Trends in antimicrobial resistance of *S. aureus* from samples tested at the University of Pretoria bacteriology laboratory, 2007–2012

Group	Antimicrobials	Resistance to antimicrobial agents by year						^a^ *P*-values of CAT Test
		2007	2008	2009	2010	2011	2012	
β-lactams	Penicillin	75.0 (6/8)	80.0 (12/15)	63.6 (7/11)	50.0 (5/10)	50.0 (3/6)	50.0 (3/6)	0.089
	Ampicillin	75.0 (6/8)	80.0 (12/15)	63.6 (7/11)	60.0 (6/10)	50.0 (3/6)	50.0 (3/6)	0.124
	Cephalothin	25.0 (2/8)	6.7 (1/15)	9.1 (1/11)	10.0 (1/10)	0.0 (0/6)	0.0 (0/6)	0.181
Aminoglycosides	Amikacin	0.0 (0/8)	20.0 (3/15)	9.1 (1/11)	0.0 (0/10)	0.0 (0/6)	0.0 (0/6)	0.258
	Gentamicin	12.5 (1/8)	20.0 (3/15)	9.1 (1/11)	0.0 (0/10)	0.0 (0/6)	0.0 (0/6)	0.098
	Kanamycin	25.0 (2/8)	26.7 (4/15)	9.1 (1/11)	0.0 (0/10)	0.0 (0/6)	0.0 (0/6)	0.017
Tetracyclines	Doxycycline	37.5 (3/8)	26.7 (4/15)	36.4 (4/11)	20.0 (2/10)	0.0 (0/6)	0.0 (0/6)	0.041
Fluoroquinolones	Enrofloxacin	0.0 (0/8)	6.7 (1/15)	0.0 (0/11)	0.0 (0/10)	16.7 (1/6)	50.0 (3/6)	0.005
	Orbifloxacin	12.5 (1/8)	20.0 (3/15)	9.1 (1/11)	0.0 (0/10)	16.7 (1/6)	33.3 (2/6)	0.718
Potentiated-sulfas	Co-trimoxazole^b^	37.5 (3/8)	33.3 (5/15)	27.3 (3/11)	10.0 (1/10)	0.0 (0/6)	16.7 (1/6)	0.067
Amphenicols	Chloramphenicol	33.3 (1/3)	20.0 (3/15)	9.1 (1/11)	10.0 (1/10)	40.0 (2/5)	33.3 (2/6)	0.552
Macrolides	Tylosin	37.5 (3/8)	33.3 (5/15)	0.0 (0/11)	10.0 (1/10)	0.0 (0/6)	0.0 (0/6)	0.008
Aminoglycoside-lincosamides	Lincospectin^c^	37.5 (3/8)	80.0 (12/15)	54.6 (6/11)	70.0 (7/10)	50.0 (3/6)	83.3 (5/6)	0.478
Lincosamides	Clindamycin	50.0 (4/8)	60.0 (9/15)	45.5 (5/11)	60.0 (6/10)	50.0 (3/6)	33.3 (2/6)	0.552
Others	Amoxicillin/clavulanic acid	37.5 (3/8)	33.3 (5/15)	9.1 (1/11)	0.0 (0/10)	0.0 (0/6)	16.7 (1/6)	0.032

^a^ *P*-values of CAT Test= *P*-value of Cochran-Armitage trend test

^b^Co-trimoxazole = Trimethoprim-sulphamethoxazole

^c^Lincospectin = Espectinomycine-lincomycine

**Fig. 3:**
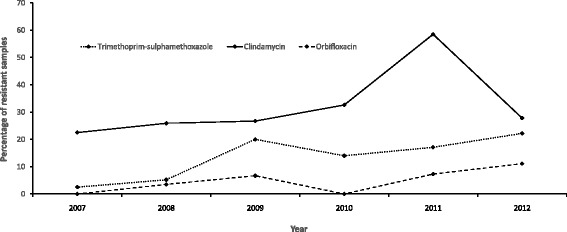
Antimicrobial agents showing significant temporal trends in resistance based on the Cochran-Armitage trend tests among the *S. pseudintermedius* isolates from canine samples tested at the University of Pretoria bacteriology laboratory, 2007–2012

The distributions of resistance among *S. pseudintermedius* isolates to the different antimicrobials is shown in Table 2. Significant increases in the proportions of resistant isolates to trimethoprim-sulphamethoxazole (*p* = 0.0040), clindamycin (*p* = 0.0221) and orbifloxacin (*p* = 0.0418) were observed among *S. pseudintermedius* isolates between 2007 and 2012 (Fig. 3, Table 2). Among *S. pseudintermedius* isolates, the proportion that were resistant to clindamycin increased until 2011 (59%) but subsequently decreased to 28% in 2012, while, the proportion of isolates that were resistant to orbifloxacin increased from 0 to 11% by 2012. Similarly, we observed an increase in the proportion of isolates resistant to trimethoprim-sulphamethoxazole from 2.5% in 2007 to 22% in 2012 among *S. pseudintermedius* isolates.

**Table 2:** Trends in antimicrobial susceptibility of *S. pseudintermedius* to antimicrobial agents from samples tested at the University of Pretoria academic veterinary laboratory, 2007–2012

Group	Antimicrobial	Resistance to antimicrobial agents by year						^a^ *P*-value of CAT Test
		2007	2008	2009	2010	2011	2012	
β-lactams	Penicillin	37.5 (15/40)	58.6 (34/58)	50.0 (30/60)	51.2 (22/43)	73.2 (30/41)	55.6 (16/36)	0.052
	Ampicillin	50.0 (20/40)	56.9 (33/58)	53.3 (32/60)	55.8 (24/43)	73.2 (30/41)	61.1 (22/36)	0.104
	Cephalothin	7.5 (3/40)	5.2 (3/58)	5.0 (3/60)	2.3 (1/43)	7.3 (3/41)	2.8 (1/36)	0.555
Aminoglycosides	Amikacin	0.0 (0/40)	13.8 (8/58)	3.3 (2/60)	0.0 (0/43)	4.9 (2/41)	2.8 (1/36)	0.382
	Gentamicin	0.0 (0/40)	10.3 (6/53)	3.3 (2/60)	0.0 (0/43)	2.4 (1/41)	5.6 (2/36)	0.777
	Kanamycin	2.5 (1/40)	3.5 (2/58)	8.3 (5/60)	4.7 (2/43)	4.9 (2/41)	5.6 (2/36)	0.612
Tetracyclines	Doxycycline	12.5 (5/40)	22.4 (13/58)	15.0 (9/60)	2.3 (1/43)	17.1 (7/41)	8.3 (3/36)	0.213
Fluoroquinolones	Enrofloxacin	5.0 (2/40)	5.2 (3/58)	6.7 (4/60)	4.7 (2/43)	4.9 (2/41)	16.7 (6/36)	0.122
	Orbifloxacin	0.0 (0/40)	3.5 (2/58)	6.7 (4/60)	0.0 (0/43)	7.3 (3/41)	11.1 (4/36)	0.042
Potentiated sulfas	Co-trimoxazole^b^	2.5 (1/40)	5.2 (3/58)	20.0 (3/60)	14.0 (2/41)	17.1 (7/41)	22.2 (8/36)	0.004
Amphenicols	Chloramphenicol	4.8 (1/21)	7.0 (4/57)	5.0 (3/60)	4.9 (2/41)	12.8 (5/36)	5.6 (2/36)	0.626
Macrolides	Tylosin	7.5 (3/40)	6.9 (4/58)	6.7 (4/60)	7.0 (3/43)	9.8 (4/41)	5.6 (2/36)	1
Aminoglycoside-lincosamides	Lincospectin^c^	22.5 (9/40)	48.3 (28/58)	26.7 (16/60)	39.5 (17/43)	58.5 (24/41)	38.9 (14/36)	0.066
Lincosamides	Clindamycin	22.5 (9/40)	25.9 (15/58)	26.7 (16/60)	32.6 (14/43)	58.5 (24/41)	27.8 (10/36)	0.022
Others	Amoxicillin/clavulanic acid	22.5 (9/40)	6.9 (4/58)	5.0 (3/60)	2.3 (1/43)	17.1 (7/41)	5.6 (2/36)	0.225

^a^ *P*-value of CAT Test = *P*-value of Cochran-Armitage trend test

^b^Co-trimoxazole = Trimethoprim-sulphamethoxazole

^c^Lincospectin = Espectinomycine-lincomycine

### Proportions of AMR or MDR among isolates of *S. aureus* and *S. pseudintermedius*

Of the 334 isolates, 80.5% (269/334) exhibited antimicrobial resistance (AMR) to at least one drug, while 28.7% (96/334) were multidrug resistant resistant (MDR). AMR was significantly (*p* = 0.0003) more common among *S. aureus* (98.2%, 55/56) than *S. pseudintermedius* (76.98%, 214/278). Similarly, MDR was also significantly (*p* = 0.0105) more frequent among *S. aureus* (42.9%, 24/56) than *S*. *pseudintermedius* (25.9%, 72/278).

**Fig. 4:**
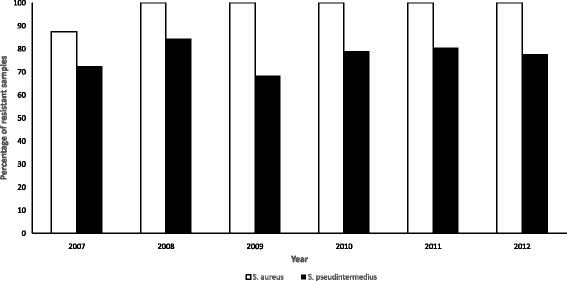
Proportions of *S. aureus* and *S. pseudintermedius* isolates resistant to at least one antibiotic from samples tested at the University of Pretoria bacteriology laboratory, 2007–2012

No significant temporal trends were observed in the proportion of AMR *S. aureus (p =* 0.1580) or S. *pseudintermedius* (*p* = 0.7312) between 2007 and 2012 (Fig. 4). However, the proportion of AMR samples was significantly (*p* = 0.0003) higher among *S. aureus* than S. *pseudintermedius.* For example, between 2007 and 2012 the proportion of AMR *S. aureus* ranged from 87.5 to 100% compared to 68% to 84% among S. *pseudintermedius* isolates.

**Fig. 5:**
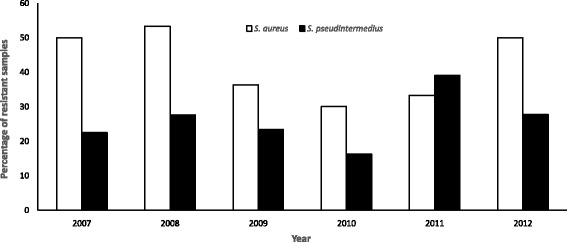
Proportions of multidrug resistant *S. aureus* and *S. pseudintermedius* isolates from samples tested at the University of Pretoria veterinary bacteriology laboratory, 2007–2012

MDR *S. aureus* were more common than MDR *S. pseudintermedius* during the period 2007 to 2012 with the exception of 2011. In 2011, 39% of *S. pseudintermedius* were MDR compared to 33% of *S. aureus* isolates. While, between 2007–2010 and 2012 the proportion of MDR *S. aureus* ranged from 53 to 30% compared to 16% to 39% among *S. pseudintermedius* isolates. No significant temporal trends were observed in the proportions of MDR S. *aureus* (*p* = 0.8212) or MDR *S. pseudintermedius* (*p* = 0.0932) (Fig. 5).

### Predictors of AMR or MDR *Staphylococcus* spp.

None of the variables investigated in this study had significant association with the log odds of either AMR or MDR.

## Discussion

Antimicrobial resistance in veterinary medicine and especially in companion animals is of increasing public health concern [2, 19]. This problem is exacerbated by the fact that the relationship between companion animals and their owners have been implicated in the cross-transmission and spread of antimicrobial resistant organisms [20–22]. Since South Africa lacks continuous assessment and reporting of veterinary related staphylococcal infections and antimicrobial resistance, the results of this study contribute significantly to improving our understanding of the trends in antimicrobial resistance among *S. aureus* and *S. pseudintermedius* clinical cases of dogs.

### Antimicrobial resistance patterns

In the present study, we observed high proportions of *S. aureus* and *S. pseudintermedius* isolates resistant to β-lactam antibiotics (ampicillin and penicillin). This is consistent with previous studies that have reported significantly higher levels of resistance of *Staphylococcus* species to β-lactam antibiotics compared to other classes of antimicrobials [23, 24]. This may be associated with the expression of intrinsic low-affinity to penicillin-binding proteins (PBPs) among *Staphylococcus* species [25] or the excessive use of β-lactam antibiotics in the treatment of *S. aureus* and *S. pseudintermedius* infections [3]. High levels of resistance to β-lactam antibiotics observed in this study will undoubtedly affect treatment and management of *Staphylococcus* spp. infections.

A significantly higher proportion of *S. aureus* than *S. pseudintermedius* isolates exhibited resistance to lincosamides (lincospectin and clindamycin). The reasons for this difference are unclear but these findings are consistent with reports of high levels of lincosamide resistance among *S. aureus* and *S. pseudintermedius* in the United States [26]. These findings suggest that lincosamides should not be used as alternative drugs in the treatment of staphylococcal infections in this population of dogs [27]. Of concern is the fact that lincosamides, such as clindamycin, have been generally known to be effective against Methicillin-Resistant *Staphylococcus aureus* (MRSA) and multidrug resistant staphylococci [28]. However, we observed that up to 59% of the *S. pseudintermedius* positive samples were resistant to lincosamides. This seems to suggest that lincosamides should not be considered for treatment of *S. pseudintermedius* infections in this population of dogs without first performing antibiograms.

### Temporal patterns

Over the study period, *S. aureus* resistance to doxycycline, kanamycin, amoxicillin/clavulanic acid and tylosin decreased significantly while resistance to fluoroquinolones among *S. aureus* and *S. pseudintermedius* positive samples significantly increased. A study conducted over a 14-year period in Canada by Prescott et al. [3] reported no significant temporal changes in *S. aureus* resistance to fluoroquinolones. Similar to findings by Pellerin et al. [5] in France among *S. pseudintermedius* isolates tested between 1987 and 1996, we observed an increasing trend in resistance to trimethoprim-sulphamethoxazole among *S. pseudintermedius*. The observed decrease or increase in antimicrobial resistance in the current study may be due to changes in usage patterns as suggested by other authors [3, 26]. Moreover, a causal link between trimethoprim-sulphamethoxazole usage and trimethoprim-sulphamethoxazole resistance in patients with urinary tract infections (UTI) has been established [29]. It has also been suggested that rapid introduction of fluoroquinolone and Trimethoprim-sulphamethoxazole drugs into companion veterinary medicine practice may also be a contributing factor to increased prevalence of resistance [30]. The authors of the present study think that this might be the case in this study. However, further investigations of the antimicrobial prescription practices among clinicians at the veterinary hospital is needed to better understand this.

Hauschild and Wójcik [4] reported 88% resistance to at least one antimicrobial drug among canine *Staphylococcus* isolates in Poland, whereas, Lilenbaum et al. [31] in Canada, reported 90.9% resistance among *Staphylococcus* spp. In our study, 80.5% of the *Staphylococcus* spp. isolates exhibited resistance to at least one antimicrobial drug. With regards to multi-drug resistance, the percentage MDR isolates in the current study was higher (28.7%) than the 24.5% reported by Gandolfi-Decristophoris et al. [32] in Switzerland and lower than the 34% as reported by Schmidt et al. [33] in the UK. The reasons for the high MDR among *Staphylococcus* spp. isolates in this study is unclear. However, we hypothesise that this may be an early indication of changes in the usage patterns among veterinarians caring for animals whose samples were included in the study. In view of this, more research needs to be done to provide a much clearer picture of the situation.

### Distribution and predictors of AMR or MDR

Almost 100% of the *S. aureus* positive samples were resistant to at least one antimicrobial agent compared to only 77% of *S. pseudintermedius* positive samples. Although resistance to at least one antimicrobial agent was lower among *S. pseudintermedius,* it was much higher than 5.2% reported as by Vanni et al. [34] in Italy and 2–40% as reported by Blunt et al. [35] in South Africa. Our findings are consistent with reports of higher levels of MDR among *S. aureus* (ranging from 51% to 67%) compared to *S. pseudintermedius* (ranging from 5% to 14%) in the UK [36] and Canada [6]. On the contrary, Jung-Ho Youn et al. [37] reported no MDR in S. *aureus* compared to 10.4% MDR among *S. pseudintermedius* in Zambia. The results of this study suggest that MDR is more common in *S. aureus* isolates than *S. pseudintermedius* from dogs presented at the veterinary academic hospital in South Africa.

### Study limitations

Hoekstra and Paulton [6] reported that the site of isolation is a risk factor of resistance. Unfortunately, we could not assess this in the current study due to the large number of categories (site types) and the small sample sizes associated with each category (site type). Some of the categories in some of the sub-analyses performed had low sample sizes and high variances hence lower precision. Previous exposure to antimicrobial agents has been associated with increased risk of development of antimicrobial resistance [20]. Unfortunately, due to the retrospective nature of the current study, the history of antibiotic use was not available. Therefore, we could not investigate the association between previous antibiotic use and antimicrobial resistance patterns. Furthermore, the study focused on canine clinical cases that were submitted to the bacteriology laboratory for diagnosis. Often such cases would include animals that have not responded well to previous treatments. Therefore, it is possible that a large population of dogs that responded to empirical treatments were not included in this study. Some sub-analyses were not possible due to relatively low sample sizes associated with some categories of some variables such as specimen type. Additionally, the time when culture and sensitivity test was done in relation to the time after hospital admission of the dogs was not available making it impossible to identify nosocomial from community acquired infections. Moreover, the geographic area covered by the study was limited to Gauteng Province which is not representative of South Africa as a whole nor is it representative of other veterinary hospitals in South Africa. These limitations, notwithstanding, the findings from this study provide useful information to guide future studies to better understand antimicrobial resistance in dogs.

## Conclusion

Antibiotic resistance among *Staphylococcus* spp. from dogs presented to the veterinary academic hospital was high and continued to increase for enrofloxacin, trimethoprim-sulphamethoxazole, clindamycin and orbifloxacin during the 7-year study period. Of concern are the increasing levels of resistance to fluoroquinolones and sulphonamides among *S. pseudintermedius.* This calls for urgent action to address the problem. The actions may include development of antimicrobial stewardship program for veterinary and para-veterinary personnel to be offered by the university as part of continuing education. Furthermore, training of veterinary students should have a strong emphasis on antimicrobial stewardship. Lastly, the need for *Staphylococcus* species characterization and request for antibiogram as part of the protocol for diagnosis and treatment of *Staphylococcus* spp. infections should be emphasized and encouraged.

## Abbreviations

AKC: American Kennel Club
AMR: Antimicrobial resistance
CAT: Cochran-Armitage trend
CI: Confidence interval
DNase: Deoxyribonuclease
MDR: Multidrug resistant resistance
MRSA: Methicillin-Resistant *Staphylococcus aureus*
OR: Odds ratio
PBPs: Penicillin-binding proteins
SAS: Statistical Analysis System
UTI: Urinary tract infection
